# Resolution of Sporadic Hemiplegic Migraine by Correcting a Cervical Spine Kyphosis Utilizing the Chiropractic BioPhysics® (CBP®) Technique: A Case Report With Long-Term Follow-Up

**DOI:** 10.7759/cureus.63774

**Published:** 2024-07-03

**Authors:** Thomas J Woodham, Jason W Haas, Miles O Fortner, Paul A Oakley, Deed E Harrison

**Affiliations:** 1 Chiropractic, Western Plains Chiropractic, Gillette, USA; 2 Research, Chiropractic BioPhysics (CBP) NonProfit, Windsor, USA; 3 Biomechanics, York University, Toronto, CAN; 4 Physical Medicine and Rehabilitation, Chiropractic BioPhysics (CBP) NonProfit, Eagle, USA

**Keywords:** migraine with aura, cervical lateral radiograph, migraine, cervical lordosis, sporadic hemiplegic migraine

## Abstract

A 19-year-old male suffered from sporadic hemiplegic migraine (SHM) for several years and experienced significant pain and disability with sensory and motor disturbances during the migraine headaches. Weakness, abnormal vision, abnormal sensation, one-sided disabling motor weakness, and other signs of SHM were diagnosed. The patient had received previous physical therapy, chiropractic and over-the-counter medications, as well as migraine-specific prescriptions without lasting improvements. Chiropractic BioPhysics® (CBP®) spinal structural rehabilitation protocols were used to increase cervical lordosis and improve cervical muscular strength, mobility, and posture. These protocols include spine-specific prescriptions for Mirror Image® postural exercises, traction, and spinal manipulative therapy. After 24 treatments over eight weeks, all subjective and objective outcomes improved dramatically with a near resolution of all initial symptoms of SHM. There were a significant increase in cervical lordosis and a reduction in forward head posture. The neck disability index improved from 26% to 6%, and all pain scores for all regions improved following treatment. A 10-month follow-up exam showed the outcomes were maintained. SHM is rare and debilitating, is part of the global burden of disease, and is a major cause of disability in the world. Reports of successful conservative and non-conservative long-term treatments for SHM are rare, and there are no clinical trials showing successful treatments for SHM. This successful case demonstrates preliminary evidence that CBP spinal structural rehabilitation may serve as a treatment option for SHM. Future studies are needed to replicate the findings from this case.

## Introduction

Migraine headache is a significant cause of daily suffering, dysfunction, lost productivity, and years lived with disability and affects up to 16% of the population [1]. Sporadic hemiplegic migraine (SHM) is a rare subtype of migraine in which the aura is characterized by the presence of motor weakness [2]. SHM, classified as 1.1.2.3.21 [3] by the International Classification of Headache Disorders, is difficult to treat. Although drug therapy is common, no long-term studies have shown a beneficial cost/benefit analysis [4-6]. Genetic, vascular, and geographical studies have been performed but are not conclusive in demonstrating the causes of SHM [2].

Chiropractic BioPhysics (CBP®) rehabilitation protocols [7-9] have previously demonstrated improvement in multiple conditions with outcomes that have been shown to be repeatable and reliable and reveal potential treatment options for physicians who treat pain and spine disorders including headache, neck pain, and migraine headache [10,11]. Indeed, a recent trial utilizing the CBP technique demonstrated that a multimodal rehabilitation program incorporating cervical extension traction showed that improvement in cervical lordosis resulted in improved headache symptoms up to two years following treatment [12]. Thus, the loss of lordosis is one biomechanical factor important in the consideration of treatment options for patients suffering from headaches [13]. 

Since the pathophysiology of SHM is limited within the wider literature, we propose that the loss of cervical lordosis may be an important biomechanical factor in the persistence of SHM symptoms. Consistent with many clinical trials [10] and case reports [11], the improvement in cervical lordosis has been shown to provide relief for many craniocervical ailments and may be effective in treating SHM. This report documents a patient with SHM who had previously had unsuccessful treatment with medications and sought an alternative therapeutic approach with a CBP® rehabilitation facility.

## Case presentation

Patient history and subjective and objective clinical findings

A 19-year-old male suffered from SHM for seven years following a trauma that resulted in a fracture at T12. The patient reported severe low back pain following a sledding injury. The focus of the imaging was on the thoracolumbar spine. He was evaluated and treated at a small hospital in rural Wyoming, United States. He was referred to a larger hospital for evaluation and treatment of the spine injury. Magnetic resonance imaging (MRI) demonstrated microtrabecular fractures with bone bruising at T12-L3 and an acute compression deformity at T12 (MRI report available upon request). The lumbar spine was treated with narcotic medications and non-steroidal anti-inflammatories.

The patient began to have headaches with aural symptoms initiating with fullness and thick sensation in the tongue which would lead to poor and slurred speech. The numbness and tingling would then spread to one side of the face. The opposite side of the face would then droop and lose motor function, further worsening the speech symptoms. The medical evaluation was performed by a small physician's office more than seven years prior to the patient receiving treatment in the facility, and the records of the initial diagnosis are unavailable. The patient's facial symptoms were initially diagnosed as Bell's palsy, but the symptoms progressed frequently to the upper extremities, and he would progressively lose sensation on one side of the torso with simultaneous contralateral motor loss. This would move from the head and neck down to the lower extremities, and the sensory and motor losses would frequently change and alternate sides.

All of this was associated with an extremely sharp pain behind the eye on the side of the sensory change unilaterally. The intense sharp head pain was severe enough to cause absolute photophobia during the event. The vision in that eye would have bright flashes, floaters, frequent loss of vision across parts of the field, and occasional complete loss of vision on the side of the eye and head pain. The patient also reported head pain that would be consistent with daily tension-type headaches occurring until receiving the treatment described in this study. The SHM headaches would happen 2-3 times per week initially and at best throughout the seven years prior to treatment were 2-4 times per month. The severity and duration of these headaches caused significant suffering and loss of productive function. Due to the long-term nature of the condition, the diagnoses of epilepsy and encephalitis were ruled out.

The patient was treated initially with narcotics for pain due to the thoracic spine injury, anti-inflammatories including ibuprofen, and, later, large doses of Excedrin migraine medicine for over seven years, and in 2017, he began to receive treatment for the lower back pain with a chiropractor performing spinal manipulative therapy. The spinal manipulation would relieve the back pain temporarily but had no effect on the daily chronic headaches and no reduction in the severity, duration, or frequency of the SHM.

The patient reported never meeting his biological father and having no knowledge of the health history of the paternal side. The patient's maternal lineage had a headache and migraine headache history with a note that his maternal grandmother suffered from occasional headaches and an episode of Bell's palsy, causation unknown. No other familial genetic tests were known to have been performed to determine if the SHM had a genetic contribution. Possibly, the initial diagnosing physicians were unaware of the genetic diagnoses or disregarded the testing due to the apparent traumatic causation.

Consultation and history found the patient had neck stiffness bilaterally and intermittently from the upper cervical spine to the upper trapezius muscles. Frequent and severe tinnitus was reported and aura-associated prior to the migraine pain. He had periodic disabling headaches that would begin with sensory abnormalities and aura and result in severe debilitating motor symptoms including bilateral and/or unilateral numbness, tingling, and aching pain reported using the quadruple visual analog scale (QVAS total 33/100) [14] causing disability in both arms. The weakness and altered sensation would cease with the cessation of the headache which ranged from 24 to 72 hours. He had these headaches 2-4 times per month, and the location of the head pain was mostly, but not exclusively, the left side of the temporal region. Concerningly, he reported that during the migraine, his lower extremities would also alternatingly become weak, numb, and tingling and would be associated with pain, disabling cramping, and motor weakness throughout his body.

A significant complaint was the cramping of muscles prior to and during SHM. During the initial evaluation, he reported cramping in the gluteus medius muscles bilaterally, bilateral thoracic paraspinal muscle cramping and spasm, and increased pain with all range of motion (ROM) testing (QVAS/thoracic pain 67/100). He also reported moderate to severe mid to low back pain frequently at the thoracolumbar junction from multiple positions. Frequently, the patient experienced gastroesophageal reflux disease (GERD), and periodic and lifelong winter-month lung infections were also reported.

Radiographic findings

**Figure 1:**
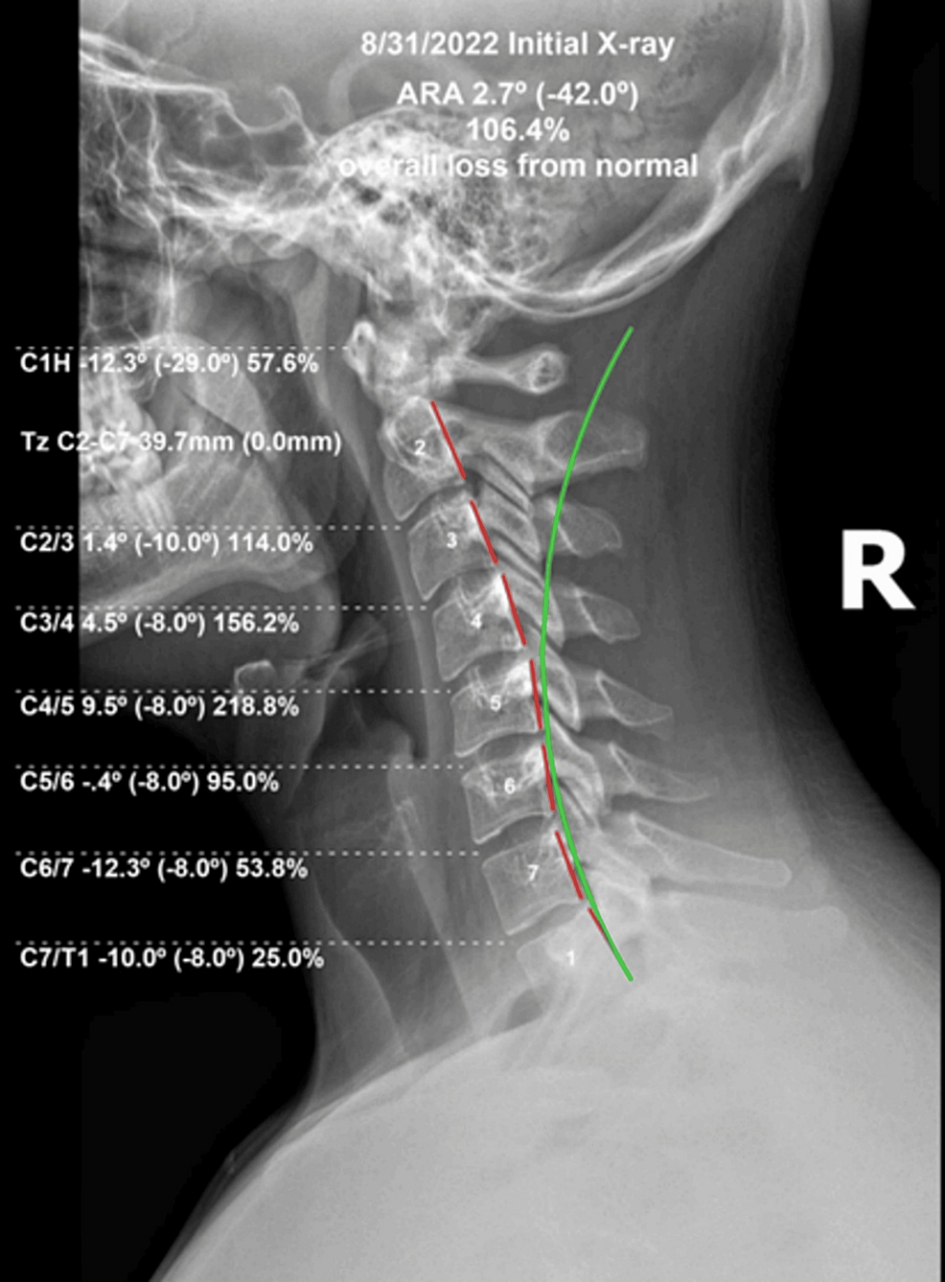
Upright, standing lateral cervical radiograph at the initial examination The ARA from C2 to C7 measures 2.7° compared to a normal range of -20° to -42°. The upper cervical spine is markedly anterior with a large abnormality visible. The green line represents normal lordosis, and the dashed red line demonstrates the posterior tangent of the segments as measured with the HPTM. The segmental angles reported on the left are the individual RRAs. These angles are the first derivative of the curvature of the column, and the greater the abnormality, the larger the stresses and strains generated on the tissues ARA: absolute rotation angle; HPTM: Harrison posterior tangent method; RRAs: relative rotation angles

Upright coronal and sagittal radiographs demonstrated abnormalities in the cervical curvature with kyphotic deformity found from C2 to C7 and measuring +2.7° vs. the ideal absolute rotation angle (ARA) of -42° [15,16]. C1 to the horizontal angle measured -12.3° compared to average-to-ideal angles of -24° to -29° [15]; the anterior translation of the head measured 39 mm compared to the average of 12-15 mm [15]. Relative rotation angles at C2-C6 were at or greater than 100% in abnormal spinal alignment with C4/C5 measuring greater than 200% of normal tolerance for joint position; these are thought to abnormally load the neural tissues [17]. See Figure 1.

Orthopedic and objective outcome measure findings

The patient reported pain from orthopedic cervical compression testing that was localized on the right side of the neck. ROM to evaluate pain found restriction with pain during flexion, and lumbar ROM testing revealed severe restriction with flexion and pain and restriction with the patient performing left lateral flexion of the torso. Sensory pinwheel testing found hypoesthesia at the C3 dermatome. The headache disability inventory (HDI) [18] measured 28% at initial evaluation. His initial neck disability index (NDI) [19] measured 26% indicating mild to moderate disability due to neck pain. He reported low back pain intermittently in the mid lumbar spine with a modified Oswestry disability index (MODI) [20] measuring 22% indicative of moderate disability due to pain. The RAND short-form 36-question health status questionnaire (SF-36) [21] indicated disability in the categories of general health (62/100), physical function (25/100), social function (63/100), mental health (76/100), bodily pain (58/100), and energy/fatigue (50/100). See Table 1.

**Table 1:** Short-form 36-question health status questionnaire results for initial evaluation, re-evaluation following treatment, and long-term follow-up This table reports the patient's health status across multiple parameters via a 36-question questionnaire. 0 would be a very poor function and 100 would be a perfect function. Health perception, physical function, physical ability, emotional health, social health, mental health, dysfunction due to bodily pain, and energy levels/fatigue are reported. Overall change represents the difference from initial evaluation to long-term follow-up

Date	Health perception	Physical function	Physical ability	Emotional health	Social function	Mental health	Bodily pain	Energy/fatigue
Initial	72	84	81	81	83	75	75	61
9/8/2022	62	85	25	100	63	78	58	50
11/4/2022	77	90	100	100	88	80	80	65
8/1/2023	52	95	100	100	100	88	80	75
Overall change	-10	10	75	0	37	10	22	25

Treatment protocols and frequency

**Figure 2:**
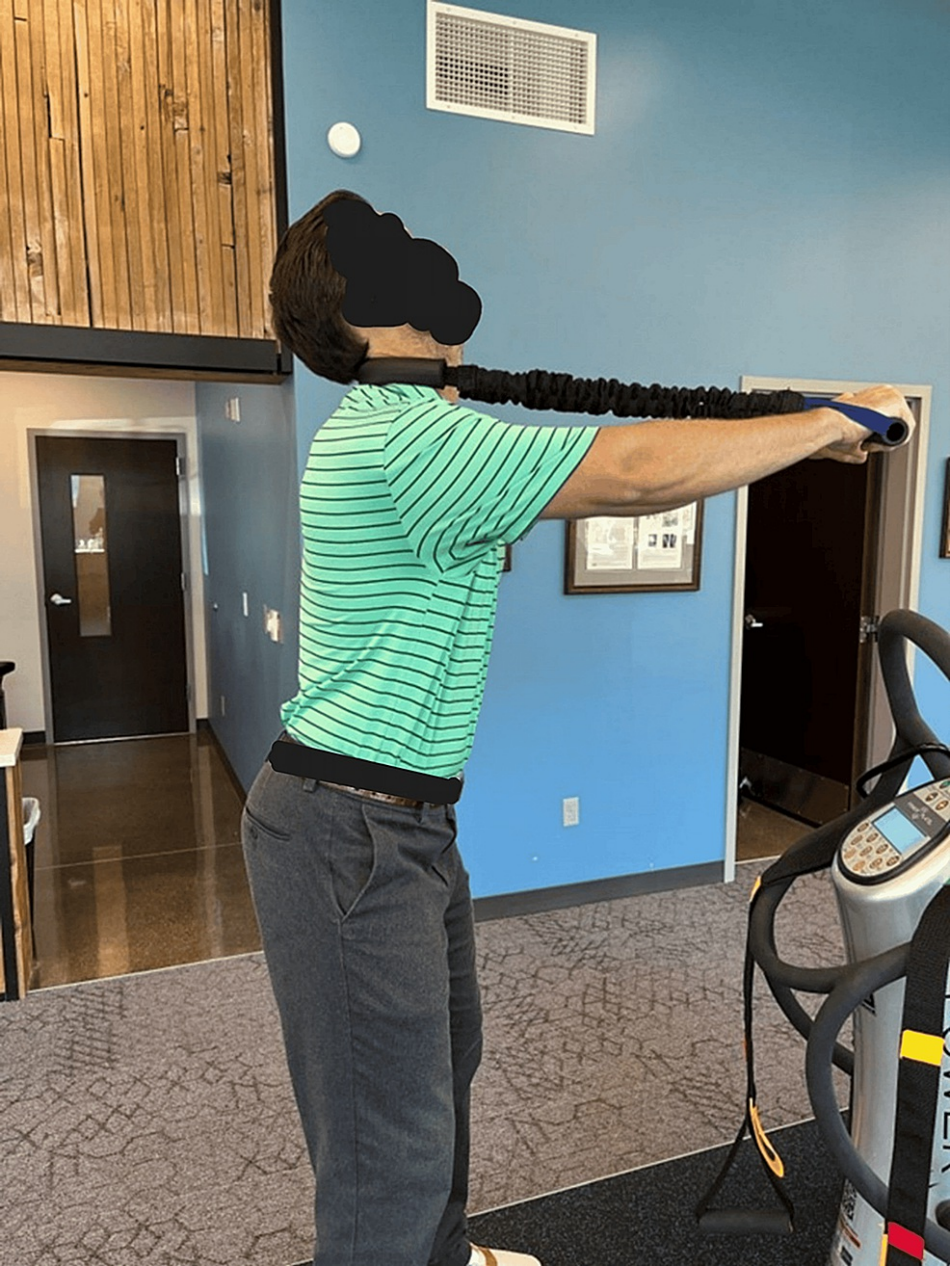
The sagittal Mirror Image® exercise using the ProLordotic® performed on the Power Plate® The patient extends over the elastic exercise device and actively contracts the muscles of the posterior neck while increasing intensity by straightening the arms. The patient is under the influence of whole-body vibration causing numerous stabilizing muscles to contract throughout the body

**Figure 3:**
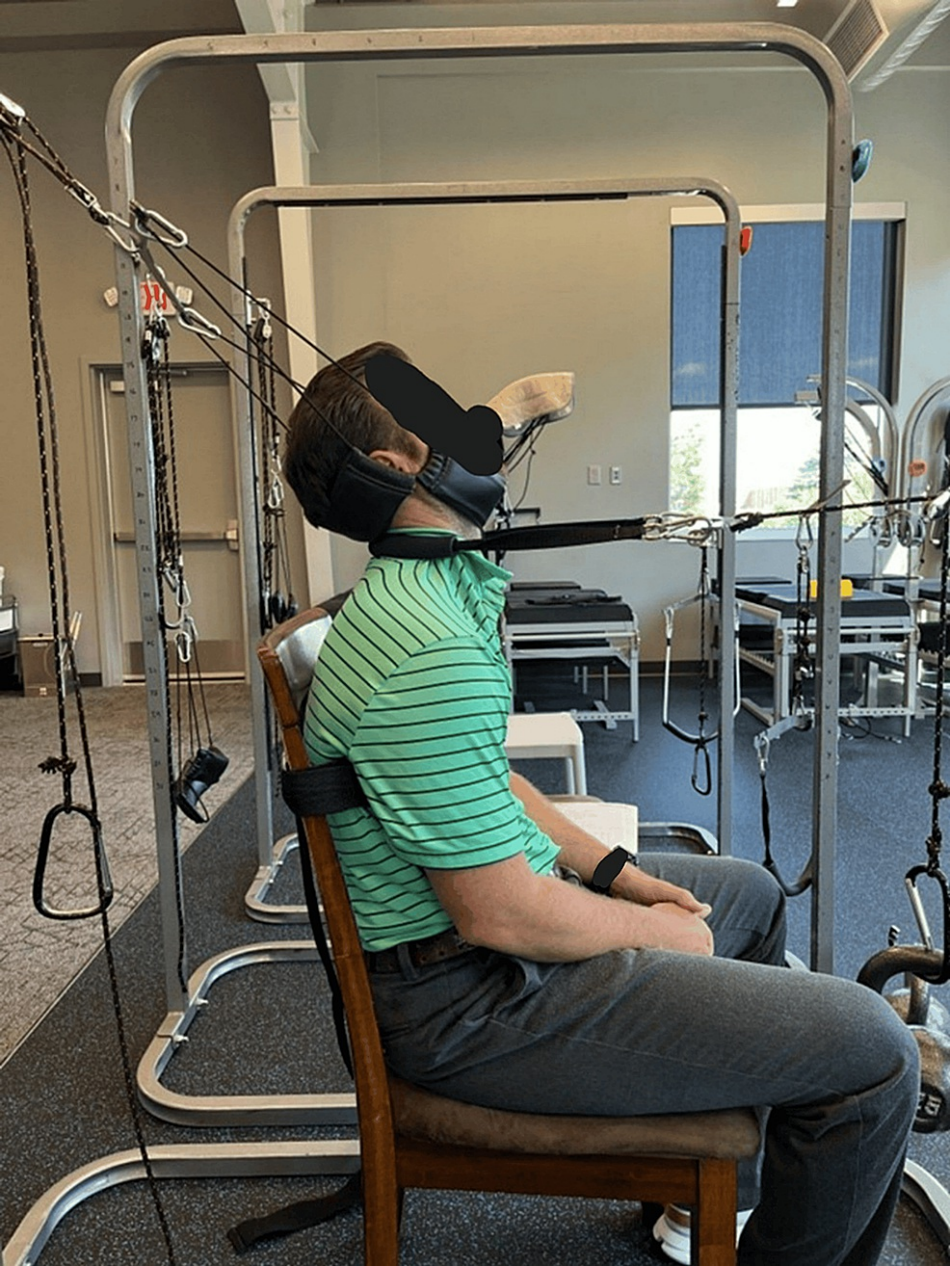
Two-way CET The patient is extended, and a slight distraction force posterior translates the head and extends the upper cervical spine, while a stronger force toward the front induces lordosis in the mid-cervical spine segments.  The chest is restricted from moving forward, and the patient exaggerates the thoracic spine to increase upper thoracic flexion and lower cervical extension. This traction is the mirror image of the radiographic findings CET: cervical extension traction

The patient received CBP® structural spinal rehabilitation protocols [7-9] in-office for 24 treatments over eight weeks. These rehabilitation methods include PostureRay radiographic documentation system machine learning modeling to allow the physician to prescribe specific Mirror Image® (14MI) exercises that are designed to strengthen the muscles of the spine that are involved with movement in the direction opposite of the spinal abnormality. Additional spine strengthening exercises using the ProLordotic™ cervical spine exerciser (Circular Traction, LLC, Huntington Beach, California, United States) were performed under whole-body vibration (WBV) on the Power Plate® [22]. See Figure 2. Furthermore, spinal traction designed to place the spine into the MI® per the PostureRay® measurements was used. This slow and gentle traction force primarily targets the viscoelastic ligamentous structures of the spine that do not have the contractile abilities of muscles and require prolonged application of forces in the correct MI® direction to attempt to correct these spinal abnormalities. Traction begins at 2-3 minutes, and the patient is progressed to as much as 15-20 minutes based on tolerance (Figure 3). Specific SMT was applied based off the radiographic abnormalities and in the direction opposite of the misalignments using a fulcrum (LSpa™ device, Manalapan, New Jersey, United States) under the posterior neck below the kyphotic deformity while an anterior-to-posterior thrust was performed. The SMT is used to increase range of motion and decrease pain.

The patient was treated in-office and was given home care including postural exercise, postural education, and the prescription of a simple device to assist in-office traction (Denneroll™ cervical traction orthotic (DCTO)). The patient was instructed to perform postural exercises 3-5 times per week for 15 seconds per repetition and to perform as many repetitions as tolerable. DCTO was initiated at 2-3 minutes and progressed to 15-20 minutes. The patient was compliant with in-office therapy as well as home care. Upon re-evaluation including spinal radiography, all outcome measures and QVAS were re-assessed. No adverse events were reported by the patient.

Results

Subjective and Objective Clinical Findings

Following 24 in-office treatments consisting of MI® exercises, traction, and postural SMT, all initial subjective and objective outcome measures were repeated. Comparison of nearly all outcome measures, both subjective and objective, found improvements from baseline data. The patient self-reported that his SHM pain and disability were improved by 90%. Paralysis symptoms including leg weakness and numbness and loss of function were resolved 100% as reported by the patient. Neck stiffness was reported completely resolved, and visual ROM analysis for pain found no abnormalities. The cramping that was initially reported had completely resolved, no orthopedic tests were positive, and none induced cramping as was found initially.

Assessment of the improvements was made by the initial examination physician and included all relevant patient interviews regarding their reported subjective improvements. The examination also included a confirmation that the patient was performing the home exercises designed to increase lordosis and improve forward head posture (FHP). Neurological tests, strength testing (manually), visual ROM for pain, sensory tests, and cranial nerve tests including pupil with bright light were used to assess normal pupil movement as well as any lingering photophobia. All orthopedic tests were negative. No aural symptoms were reported, and the SHM appeared to have nearly completely resolved. Tension-type headache symptoms were nearly resolved and would only occasionally increase with stress and dehydration. The lordosis-inducing exercises and home traction were reported to alleviate any head pain and headache symptoms rapidly. SF-36, NDI, HDI, pain assessments, radiographs replicating the initial assessment, and all initial tests were reproduced with improvement found in both subjective and objective outcome measures as reported below. 

Radiographic Outcomes

**Figure 4:**
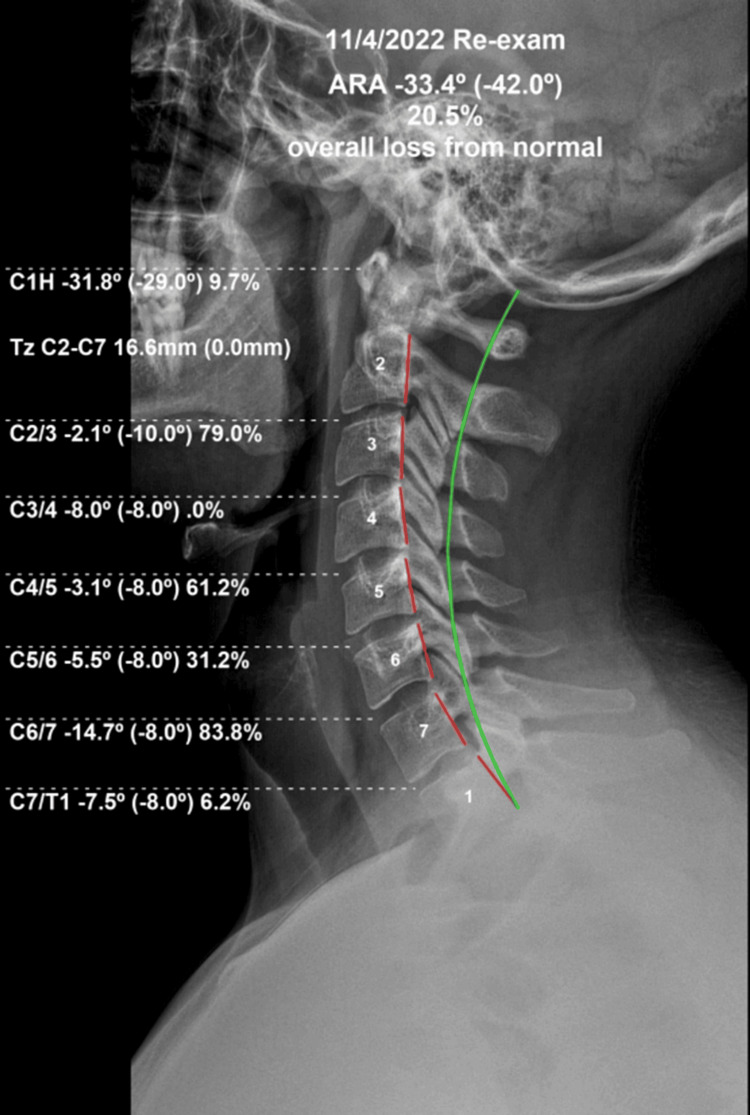
Re-evaluation upright lateral cervical radiograph Following 24 treatments in-office with home therapies according to CBP® protocols, a much improved deep cervical lordosis is visualized. The green line represents an ideal lordosis, and the red dashed line measures the posterior tangents of the segments. Values within parentheses are ideal, and values outside the parentheses represent the patient's parameters as measured with PostureRay®

**Figure 5:**
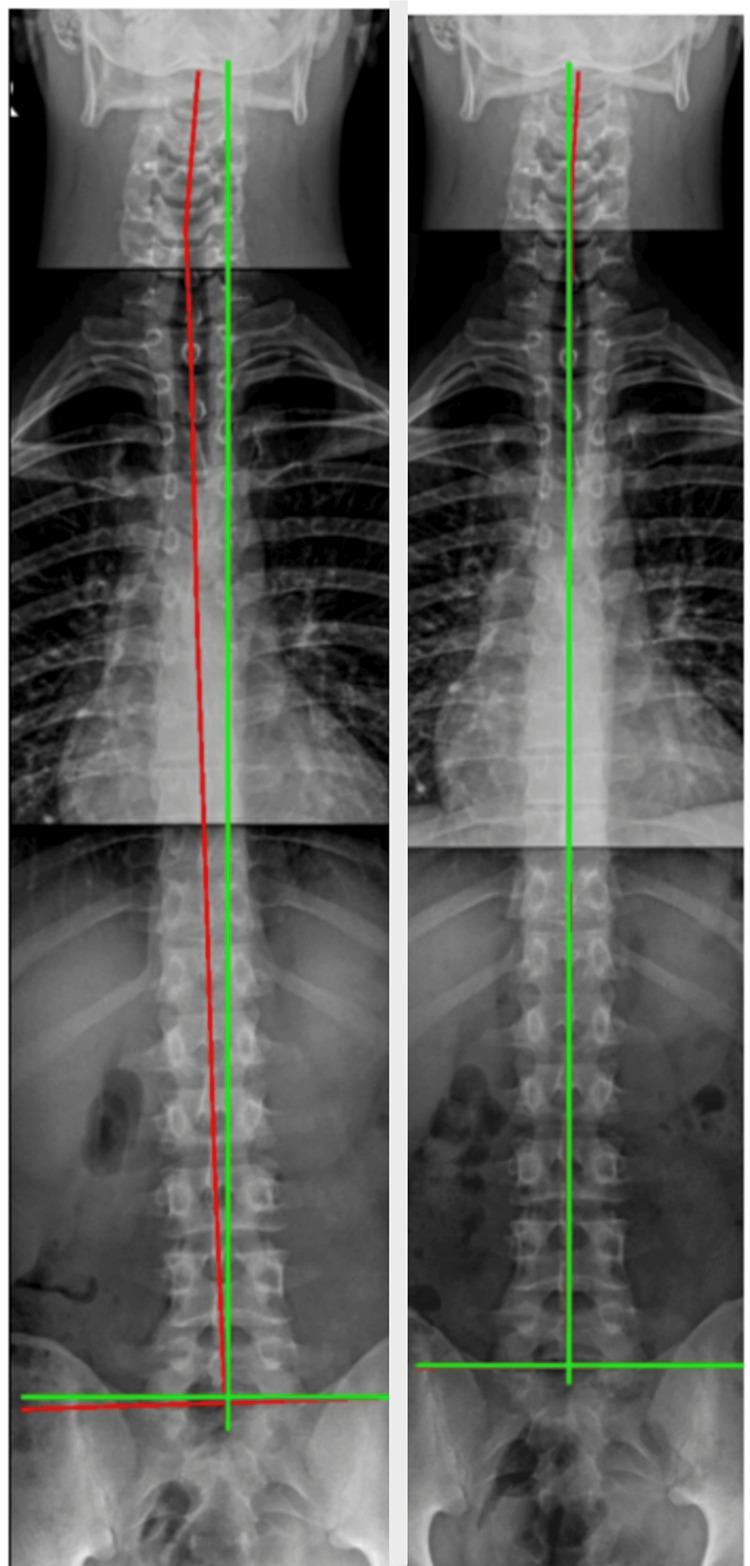
Full-spine sectional stitched anterior-posterior radiograph The initial evaluation and follow-up anterior-posterior radiograph demonstrated post-treatment improvement in the coronal balance and spine alignment. The red line represents a centroid measurement of the vertebral bodies before treatment on the left and post-treatment on the right. The green line represents the ideal skeletal coronal and spine alignment

A follow-up examination was performed more than 24 hours following the previous treatment to prevent any possible lingering effects of that treatment. Follow-up radiography found the ARA in the cervical spine from C2 to C7 was significantly improved from an initial measurement of +2.7° and the re-assessment ARA measured -33.7° with a normal range from -34° to -42°. Positive translation of the head compared to the thorax was significantly improved from the initial finding of +TzH of 39.7 mm to 16.6 mm with normal being 0 mm. The atlas plane line was improved from an initial measurement of -12.3° to -31.8° with a normal range from -24° to -29°. See Figure 4. The anterior-posterior (A-P) stitched full spine films showed dramatic improvement in coronal balance at re-exam (Figure 5).

Follow-Up Findings

A 10-month follow-up assessment was performed following in-office treatment performed one time per month and DCTO use performed 2-3 times per week at home. The patient continued home exercises. At assessment, all initial examinations and subjective and objective outcomes were repeated. The patient continued to have no SHM symptoms and no reported symptoms that caused him to seek care initially and reported his satisfaction with the in-office and home care was very high.

**Figure 6:**
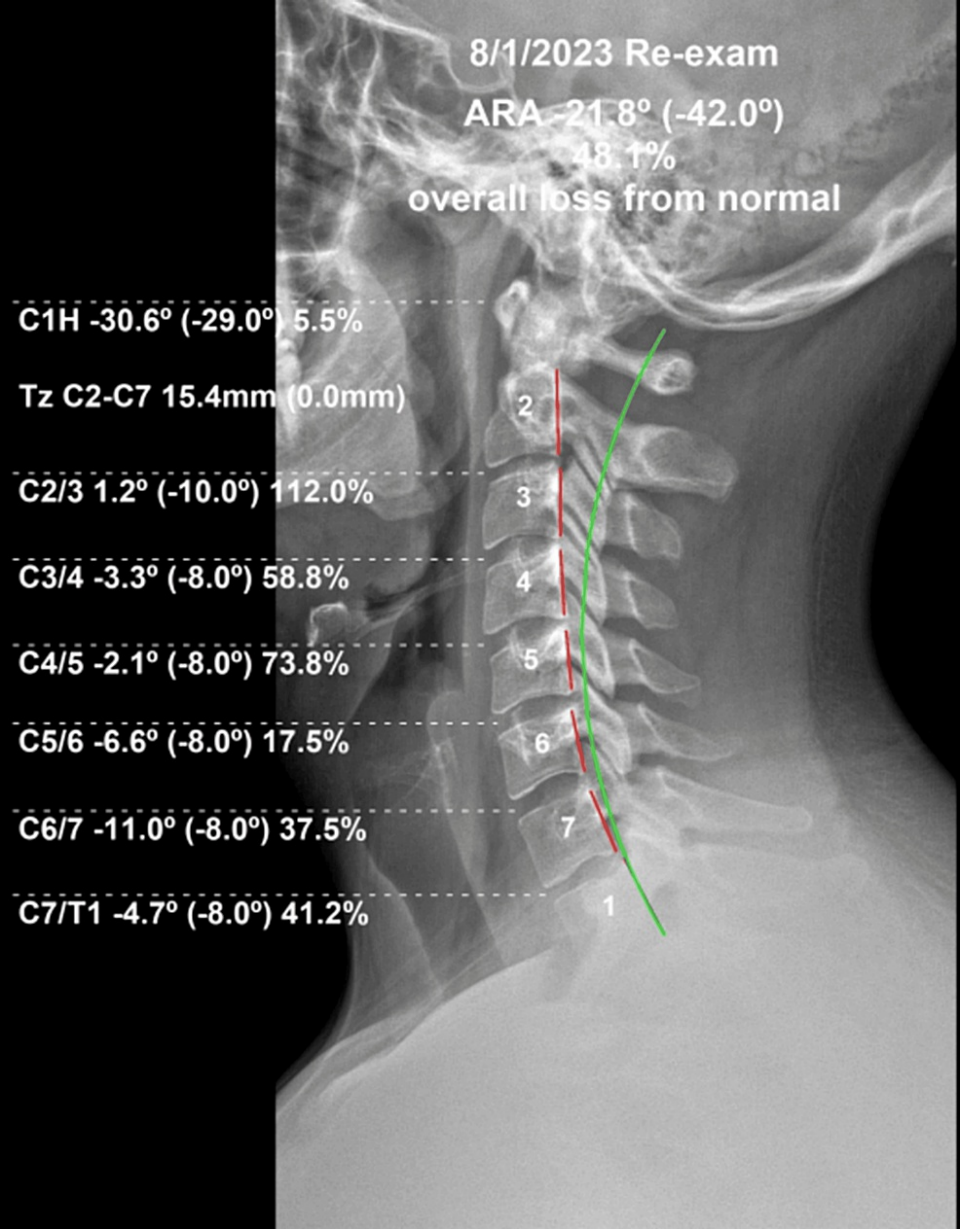
Ten-month follow-up lateral cervical radiograph Long-term lateral cervical radiograph demonstrates sagittal balance is well maintained and the lordosis is stable with a regimen of one in-office treatment per month and consistent home Denneroll® cervical orthotic use. The red line is the patient's posterior tangents, and the green line represents an ideal mathematical lordotic model

Lateral cervical radiograph revealed the lordosis was maintained compared to prior reassessment; however, the overall ARA had reduced to -21.8° which is still above the "normal" cervical cut-point of -20° [16]. Near-normal FHP remained [15], measuring 15.4 mm compared to the prior evaluation measurement of 16.6 mm (Figure 6). Cervical QVAS found very minimal discomfort with the initial measurement at 33/100, the re-assessment measuring 13/100, and the 10-month follow-up showing 17/100 for mild neck pain infrequently. NDI improved from 26% to 6% at re-assessment and was maintained (4%) at follow-up. Importantly, the QVAS for low back pain measured 63/100 initially with a significant reduction in pain to 20/100 at re-assessment and continued improvement at long-term follow-up of 7/100. This is significant because the treatment was directed primarily to the cervical spine and posture. SF-36 assessment found continued improvement in quality-of-life categories (Table 1). HDI overall improvement was measured from 28% initially to 6% at re-assessment. SF-36 assessment was found to have significant improvement across all categories with the most significant change found in physical function with 100/100 points improved compared to initial assessment.

## Discussion

This case documents a patient with the rare condition of SHM having significant and measurable improvement in patient-reported outcomes (PROs), health-related quality of life (HRQoL) measures, and radiographic changes in spinal alignment with CBP® protocols. Measured spinal abnormalities and altered alignment appear to have played an important part in the patient's non-improvement with prior pharmacological treatments [13,23,24]. Loss of cervical lordosis has been discussed as a potential causative factor in the worsening of the duration and intensity of cervicogenic and other headaches. There are no known conservative studies previously reported showing improved outcome measures using CBP® rehabilitation protocol or other physiotherapeutic or manual therapies in SHM patients.

SHM is rare in the literature, and there are no randomized controlled trials (RCTs) published regarding effective treatment options. The SHM symptoms are very concerning to the patient as they frequently mimic cerebrovascular accident (CVA) and often require significant differential diagnosis [1,4]. Since the altered motor and sensory symptoms reverse after the episode subsides, many patients are aware of the aura that precedes the cephalgia and is then followed by the abnormal numbness, tingling, weakness, and altered visual and auditory and vocal symptoms that can frequently change sides with episodes. Our patient presented similar symptoms. The patient in our case reported a familial history of migraine with aura but did not undergo genetic testing. Physical examination and history are crucial to the diagnosis of SHM, and our patient met the criteria for the diagnosis of SHM. Starting with aura symptoms that preceded the cephalgia, bilateral and/or unilateral head pain would follow with motor weakness, visual disturbances, and sensory and processing abnormalities that would resolve with headache cessation [4,25]. Our patient did not have other ICD-10 diagnostic criteria that would have accounted for the symptoms and episodes.

There are some studies in the literature that report positive outcomes with drug therapy including NSAIDs as well as studies involving prescription flunarizine, ketamine, naloxone, and lamotrigine [26]. Prednisone and steroids are reported as well as valproic acid, tryptamine, and topiramate [27]. These drugs have not been studied in large RCTs for use in SHM patients and are mostly anecdotal in nature, and no studies report any long-term conclusions that the drugs are successful as a lifetime treatment.

No conservative non-drug studies in the medical literature documenting the successful treatment of SHM with long-term follow-up and high-quality HRQoL measures were found [26]. The pathoanatomical and pathophysiological mechanisms of SHM are not known due to the rarity of the condition. However, there have been investigations previously discussing pathoanatomic loads occurring with loss of lordosis and poor outcomes including severe migraine. The effect of mechanical and pathological forces on human nervous tissue has been investigated since 1882 by Symington [28]. He measured the strength and failure forces on many different types of nerves [28]. Breig [29] as early as 1960 and more extensively in 1978 discussed the abnormal loads and nervous system mechanical tension that is created when the cervical lordosis is lost [30]. He found that adverse (i.e., pathologic vs. physiologic) tensions were created with hypolordosis of the cervical spine, specifically in cranial nerves 5-12, the brainstem, the spinal cord, and peripheral nervous tissues, and that these pathologic loads were implicated in overt symptomatic expression, that is, resulted in neurologic consequences. Numerous adverse neurological consequences of loss of lordosis including headache, migraine, spine pain, loss of function, disability, and central and peripheral symptoms and disorders have been reported [13,28-32].

Other animal models have confirmed that nervous system tissues require an optimal balance between tension, compression, shear, and torsion. When the tissues are overloaded and the balance between the forces is lost, pathoanatomic, hemodynamic, and neurological consequences will follow [33,34]. The spinal column has alternating curvatures that, with movement, enable the corresponding forces exerted onto the nervous tissues to be minimized, that is, avoid adverse stresses and strains. Further, Breig and others studied the effect of increasing lordosis via surgery and conservatively to improve hemodynamic microvascular integrity, reduce abnormal tissue loading, and lessen the likelihood of further progression of the nervous system pathophysiological mechanisms that lead to pain and dysfunction [29,30,33-35].

Given this historical understanding of the necessity of cervical lordosis to normal function and reduction in dysfunction and degeneration, a correlation between lordosis and SHM can be made in this situation where the significant forces in the sledding crash likely led not only to thoracolumbar compression fracture but also snap-through buckling and reduction of the cervical lordosis. Given the rapid onset of the SHM following the sledding spine injury, the SHM was caused either by the abnormal mechanical tension in the central and peripheral or the hemodynamic changes that could have led to the symptoms following the spine buckling. Cervical spine alterations have long been reported to contribute to head and neck pain, but SHM as a consequence of cervical hypolordosis has not been previously reported. The genetic contribution in this case is not known and may be a limiting factor in the absolute of a "textbook"-specific diagnosis of SHM; however, the rarity of the condition makes scrutiny of the condition beyond the current literature difficult.

Clinicians and therapists should adopt economical, safe, and effective therapies for complex diseases based on the best available evidence. Since SHM is a rare condition, clinical trials are not available; therefore, quality case reports and lesser forms of evidence become more important. Thus, this case may offer physicians a potential therapeutic approach with promising preliminary results.

## Conclusions

This case report is the first successful non-drug treatment of sporadic SHM published. Migraine is found in a significant population, and SHM is a rare subset condition that has significant implications for the patient in loss of quality of life and ability to work and contributes to the global burden of disease. Treatment options are few beyond medication, and this study adds evidence towards a non-pharmacologic, manual, and conservative therapy-based approach to SHM with potentially beneficial effects. Future studies are necessary to confirm these findings and further assess if correcting cervical lordosis is an effective treatment intervention for patients with SHM.
